# Sustainable mechanochemical synthesis of β-cyclodextrin polymers by twin screw extrusion

**DOI:** 10.1007/s11356-021-15187-5

**Published:** 2021-08-23

**Authors:** Alberto Rubin Pedrazzo, Francesco Trotta, Gjylije Hoti, Federico Cesano, Marco Zanetti

**Affiliations:** 1 Department of Chemistry, University of Torino, Via P. Giuria 7, 10125 Torino, Italy; 2 ICxT Centre, University of Torino, Lungo Dora Siena 100, 10153 Torino, Italy

**Keywords:** Cyclodextrins, Biopolymers, Mechanochemistry, Twin screw extrusion

## Abstract

Cyclodextrin nanosponges (CD-NS) are cross-linked cyclodextrin polymers characterized by a nanostructured three-dimensional network. CD-NSs in the last years found many different applications in the pharmaceutical field for the controlled release of drugs and for the absorption of undesired substances from physiological media, food, and wastewater. Most of CD-NS syntheses involve the solubilization of the chosen CD in closed batch, using a suitable organic polar aprotic liquid, which may affect potential environmental or biomedical applications. Since the research is now moving towards more sustainable approaches, new and greener syntheses of CD-NS are now being developed. Here, it is reported a new eco-friendly and efficient synthesis of nanosponges through mechanochemistry. Mechanochemistry involves the application of mechanical forces to drive and control chemical reactions by transferring energy to chemical bonds. The mechanochemical approach involves the use of a twin-screw extruder (TSE) as a chemical reactor: TSE are capable of fine temperature control and, furthermore, TS Extrusion is a continuous process and not a batch process. Among the many available CD-NS syntheses, we tested our solvent-free approach on a β-CD/citric acid (CA) system. Moreover, using TSE, the same polymer was obtained in a considerably shorter time. The so obtained NSs were used for the adsorption and removal of probe molecules, in comparison with NSs prepared by cross-linking β-CD with CA in batch.

**Graphical Abstract:**

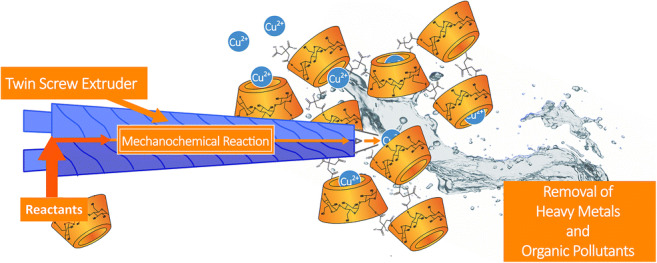

**Supplementary Information:**

The online version contains supplementary material available at 10.1007/s11356-021-15187-5.

## Introduction

In the last decades, polysaccharides and starch derivatives such as cyclodextrins (CDs) have become relevant in the wide field of nanomedicine and nanotechnology since they are safe, low cost, and biodegradable. Among them, cyclodextrin-based nanosponges (CD-NSs) have emerged due to the properties attributable to their peculiar structure (Krabicová et al. 2020). Cyclodextrin nanosponges are cross-linked cyclodextrin polymers characterized by a nanosized three-dimensional network. The reactive hydroxyl groups of CDs allow them to act as polyfunctional monomers, permitting them to be cross-linked with various multifunctional chemicals, such as dicarboxylic acids, dianhydrides, and diepoxides. It is moreover possible to tune polarity and size of the polymer network by varying the type of cross-linker and degree of cross-linking, influencing as a result the final properties (Trotta 2011; Caldera et al. 2017).

Nanosponges demonstrated in the last 20 years remarkable results in removing organic compounds and cations from wastewater (Trotta 2011; Caldera et al. 2017;), purifying for example water contaminated by persistent organic pollutants (such as chlorobenzenes and chlorotoluenes (Trotta 2011)) and found many different applications in the pharmaceutical field as drug delivery systems (Cavalli et al. 2006; Trotta et al. 2012, 2014, 2016; Swaminathan et al. 2016), showing an improvement in bioavailability and release kinetics. The CD-NSs interesting capability of hosting a wide range of different drugs goes hand in hand with their biocompatibility.

The drawbacks of these biopolymers are mainly related to the synthetic procedure. The most common CD-NSs synthetic pathway consists in dissolving the chosen CD in a suitable solvent, under continuous stirring, and then adding the cross-linker and, if necessary, a catalyst. The solvents are usually organic polar aprotic liquids, for example, N, N-dimethylformamide (DMF) or dimethylsulfoxide (DMSO).

The presence of solvents influences the entire synthesis: the final material needs to be accurately washed by an extraction with an excess of water or by the use of volatile solvents (acetone, ethanol) in order to remove the solvent inside the structure of the NS. This is essential for all possible application of nanosponges both in the biomedical and environmental field.

Furthermore, the batch synthetic procedure is not convenient for a subsequent scale up of the reaction, since huge amounts of solvent have to be disposed of. Thus, many organic solvents are expensive and, for example, solvent such as DMSO and DMF are hard to recycle because of their high boiling point. The current dependence on solvents is nowadays unsustainable for many reasons because it depends on fossil derivatives, is environmentally problematic, and is expensive and energy demanding for what concerns solvent production, recycling, and disposal. Chemical processes, according to the Green Chemistry Principles (Anastas and Warner 1998), requires nowadays to be designed in order to “minimize the quantity of final waste and to avoid hazardous or toxic solvents”. From this specific point of view, nanosponges themselves, that are synthesized from starch derivatives and biodegradable, are a very promising material.

In this article a new, green, synthesis of nanosponges through mechanochemistry is proposed.

Mechanochemistry involves the application of mechanical forces to drive and control chemical reactions by providing the energy necessary to react to chemical bonds.

Mechanochemistry has been historically a marginal approach to chemical but recently is becoming a more mainstream technique (Takacs 2013) because can promote reaction between solids quickly and quantitatively (James et al. 2012).

The application of mechanochemistry to inorganic chemistry is nowadays established and easily transferrable to an industrial scale (Burmeister and Kwade 2013; Wang et al. 2015) but in recent years also the use of mechanical forces to drive and control organic reactions and polymers synthesis has gained growing interest (Willis-Fox et al. 2018; Lanzillotto et al. 2015; Andersen and Mack 2018; Tan and Friščić 2018; Bose and Mal 2019). Moreover, esterification and etherification of starch and the possibility of obtaining CD derivatives have been recently reported (Jicsinszky et al. 2017a).

Recently, we demonstrated the possibility a CD-NS, having the same characteristics as cyclodextrin-based polymers synthesized in batch, to be obtained without the use of any solvents, via a mechanochemistry-driven reaction in ball mill (Rubin Pedrazzo et al. 2020; Trotta and Rubin Pedrazzo 2021). The reaction reported is completely carried out by mixing/grinding the dry CDs powder with the cross-linker, 1,1-carbonyldiimidazole (CDI). CDI is very reactive, and the reaction is exothermic. Generally, ball milling and grinding do not allow an accurate temperature control during the reaction. The milling jar internal temperature can even reach 200 °C in certain conditions (Andersen and Mack 2018)(Crawford et al. 2017) using for example an high-energy ball mill; however, in the tested condition for CDs and NSs, the temperature never exceeded the 72 °C (Jicsinszky et al. 2017b; Rubin Pedrazzo et al. 2020). The possibility of using refrigerated (cryomills) or preheated jars does exist, if an high temperature is undesired, but is not widely established (Crawford et al. 2017).

Furthermore, two more drawbacks affect ball mill mechanosynthesis: the first one in the use of a closed vessel for the reaction. The most commonly used reaction jar in ball mill is a closed alumina or metal vial: this is of course undesired in many organic and polymerization reactions, for example for polycondensations, where the kinetic of the reaction is dramatically slowed down by the impossibility of removing the water in the reaction batch. The other drawback is the scalability of the reactions: the maximum quantity at lab scale is in the order of grams (Burmeister et al. 2014). At industrial scale, ball mills are widely used, also on tons scale, but only for material grinding or processing. Concerning chemical reactions, the situation is completely different: higher speeds, compared to classic grinding, are usually needed for the reaction to occur and often high pressure in the jars are involved.

An innovative mechanochemical approach that could overcome these drawbacks involves the use of a twin-screw extruder (TSE) as a chemical reactor.

Twin-screw extruders are extensively used in polymer, food, and pharmaceutical industries and are a quite common lab equipment.

Recently, TSE has shown great potential in the continuous mechanosynthesis of many different preparations such as metal organic frameworks (MOFs) and deep eutectic solvents (Crawford et al. 2016, 2017). Moreover, TSE is extensively used for the reactive extrusion of polymers and for polymers functionalizations (via living polymerization or polycondensation) (O’Brien et al. 1990; Cintas et al. 2020).

TSE, indeed, are capable of fine temperature control and, additionally, TS extrusion is a continuous process and not a batch process (Andersen and Mack 2018): the use of TSE permits to move from the small scales of ball mill to a kg-scale continuous mechanosynthesis (Crawford et al. 2016).

Among the many available CD-NS syntheses, we tested our solvent-free approach on a β-CD/citric acid (CA) system. Citrate-based biomaterials, possessing tunable mechanical properties and degradation rate, can be found in many different applications (Ma et al. 2018) and are particularly promising for the environmental field.

The traditional synthesis is carried out in heated vessel under vacuum, using water as solvent (Rubin Pedrazzo et al. 2019). The polycondensation leads to an insoluble cross-linked polymer. The use of a mechanochemical approach, using TSE, permitted us to obtain the same polymer in a considerably shorter time and without using a vacuum pump.

Moreover, the use of a continuous screw system is particularly interesting for a possible scale up, a necessary condition for certain applications, such as water remediation, where large volumes of material are involved. The so obtained NSs were characterized with different techniques and then tested for the adsorption and removal of Cu^2+^ (CuSO_4_ solutions) and methylene blue (MB) from aqueous solutions, in comparison with NSs prepared by cross-linking β-CD with CA in batch using the “classic” method.

## Experimental

### Materials and methods

All the chemicals used in this work were purchased from Sigma-Aldrich (Steinheim, Germany) and used without further purifications, except for β-CD, which were provided by Roquette Frères (Lestrem, France) and maintained in oven at 100 °C up to constant weight, before use. Ultrapure water used for absorption tests was purified with a Sartorius Arium® pro ultrapure water system, 0.2-μm filtered, having a resistivity of 18.2 MΩcm,

Twin-screw extruder used for all solvent-free syntheses is Haake MiniLab II microcompounder with screw configuration (benchtop scale instrument).

Thermogravimetric analyses were performed on a Hi-res Q500 Thermogravimetric Analyzer from TA Instruments. TG analysis parameters are as follows: nitrogen flow, ramp rate 10 °C/min, from r.t. to 700 °C.

The thermograms were elaborated using TA Instruments Universal Analysis 2000 software (version 4.5A) (New Castle, DE, USA).

IR spectra on dried powders were recorded on a PerkinElmer Spectrum 100 FT-IR Spectrometer with 16 scans.

Solvent extraction for purifying samples was carried out using a pressurized solvent extractor (PSE) SpeedExtractor E-914 from Buchi.

Planetary Ball Mill is as follows: Retsch PM200 High Speed Planetary Ball Mill, 20 sintered zirconium oxide balls of 10 mm diameter in 2 jars of 50 mL (10 balls each jar), also in zirconium oxide. Milling parameters are as follows: sun wheel speed 350 rpm for 30 min, changing rotation from clockwise to anticlockwise each 10 min.

Zeta Potential and DLS measure were performed on Zetasizer Nano ZS from Malvern Panalytical. All the measurements were performed in triplicate.

FE-SEM micrograms obtained on a FESEM TESCAN S9000G, FEG Schottky source, on Cr metalized powders (5 nm Cr).

BET analysis performed on ASAP2020 from Micromeritics.

### Batch synthesis of βcyclodextrin/citric acid polymers

For the preparation in batch of cyclodextrin nanosponges: 20.00 g of β-CD, 3.73 g of sodium hypophosphite monohydrate, and the appropriate amount of citric acid for respecting the molar ratio (27.09 g for 1:8, 13.51 g for 1:4) were solubilized in 100 mL of deionized water. Then, after complete solubilization of all reactants (the solution was sonicated and heated to speed up the procedure), the solution was poured in a 20-cm-diameter crystallizing dish and heated in oven (Memmert VO500) for 1 h at 140 °C and 14 h at 100 °C, under low pressure (~ 20 mbar, oven equipped with a KNF membrane pump). After 4 h, a rigid sponge-like bulk was obtained. The obtained bulk was crushed; then, it was stirred in water.

Then, the suspension is then left to settle, and the supernatant is removed and replaced with fresh deionized water. This cycle was repeated five–six times, until a clear and colorless supernatant was observed. Eventually, the NS was filtered in a Buchner funnel, using an excess of water and acetone and dried at R.T. The dried samples were then ball milled (30 min at 350 rpm, particle size ≈ 800 nm).

### Twin-screw extruder synthesis of βcyclodextrin/citric acid polymers

The preparation of a CD Nanosponges using a twin-screw extruder involves the insertion of the physical mixture of the reactants in the correct molar ratio inside the instrument: 5.00 g of β-CD, 3.00 g Citric Acid, 0.510 g NaPO_2_H_2_·H_2_O. The quantities inserted in the extruder are calculated to fill the blending chamber and the screws section of the instrument and correspond to about 10 mL (volume).

The instrument is preheated at 150 °C, and the solid mixture is slowly inserted inside the instrument. The reaction occurs after 15 min.

After cross-linking a dried powder is obtained. The powder is extruded or recovered from the recycle chamber of the TSE. The obtained powder was stirred in water, and left to settle; the supernatant is removed and replaced with fresh deionized water. As for the batch synthesis, the cycle was repeated; then, the NS was filtered in a Buchner funnel, using an excess of water and acetone and dried at R.T. The dried samples were subsequently milled for 30 min at 350 rpm; particle size measured by DLS ≈ 800 nm, before all characterizations and comparisons with NSs were obtained from batch.

### Swelling measurements

The swelling kinetics of βCD-CA NSs was investigated by following their water uptake as a function of cross-linker content, as previously published in the literature (Hoti et al. 2021). The dry powder (0.5 g) was immersed in deionized water (in 12-mL test tubes filled up to 10 mL) for several hours until the equilibrium swelling was reached. After each predetermined time, the surface water was soaked up with tissue paper and the swollen mass was weighed. The surface water was renewed after each weight recording. The swelling rate in percentage (%S) or the water absorption capacity (%WAC) was quantified using the following Eq. (1) (Hoti et al. 2021; Afinjuomo et al. 2019; Wintgens et al. 2015; Ben Ammar et al. 2018):

1 $$ \mathrm{WAC}\left(\%\right)=\frac{m_t-{m}_o}{m_0}\times 100 $$

where m_t_ represents the weight of the swollen sample at time t and m_0_ represents the initial weight of the dry sample.

### Cross-linking density determination using Flory-Rehner theory

The swelling study was performed following the procedure described in the literature (Hoti et al. 2021). A weighed amount of about 500 mg of β-CD:CA was allowed to swell in 10 mL of deionized water (12-mL test tube) to reach equilibrium. The measurements were performed in triplicate. The parameters measured at equilibrium enabled the cross-linking density (υ) and molecular weight between cross-links (M_c_) calculation using the following Flory-Rehner Eq. (2) (J. Flory 1953; Cesar Hernandez-Ortiz and Vivaldo-lima 2013; Hoti et al. 2021):

2 $$ {\mathrm{M}}_{\mathrm{c}}=\frac{\rho_p}{v}=\frac{\frac{V_1}{\overset{\sim }{\mathrm{v}}}\left[\right.\left({\upupsilon}_{2\mathrm{m}}\right)\hat{\mkern6mu}1/3-\frac{2}{f}{\upupsilon}_{2\mathrm{m}}}{-\left[\ln \left(1-{\upupsilon}_{2\mathrm{m}}\right)+{\upupsilon}_{2\mathrm{m}}+\upchi 1{\left({\upupsilon}_{2\mathrm{m}}\right)}^2\right]} $$

where υ_2m_ is the polymer volume fraction in the equilibrium-swollen polymer, ρ_p_ is the polymer density, V_1_ is the molar volume of water as a swelling agent, χ_1_ is the Flory-Huggins solvent-polymer interaction parameter, ṽ is the specific volume of polymer, and f is the functionality of cross-links.

### Rheological measurements

Rheological measurements were carried out in a Rheometer TA Instruments Discovery HR 1 modifying the procedure as detailed in the existing literature (Hoti et al. 2021). The 20-mm diameter roughened surface geometry such as crosshatched plate and 20-mm diameter stainless-steel plate geometry were employed to enhance the contact between the sample and geometry. The prescribed gels were placed between the stationary surface and upper parallel plate with a 0.3 mm (20-mm diameter stainless-steel plate geometry) and 1 mm (20-mm diameter roughened surface geometry). The gels were examined by the oscillatory shear mode to determine the viscoelastic region. The storage modulus (G’) and the loss modulus (G”) were accessed by performing frequency sweep measurements from 100 to 0.2 rad/s and constant stress amplitude of 2%. The measurements were accomplished in triplicate recording their average. The storage modulus (G’) determined by the performance of the rheological measurements enabled the calculation of the number of elastically effective chains per unit volume (υ_e_) as presented by the following Eq. (3) (Calvet et al. 2004; Hoti et al. 2021):

3 $$ \mathrm{G}{'}_{\mathrm{p}}=\left(1-\frac{2}{f}\right)\times {\upupsilon}_{\mathrm{e}}\times \mathrm{R}\times \mathrm{T} $$

υ_e_ is the number of effective chains per unit volume estimated in mol cm^−3^, R is the universal gas constant (8.314 J mol^−1^ K^−1^), T is the temperature, and f is the functionality.

### Adsorption of Cu^2+^ ions (≈ 500 ppm)

A Cu^2+^ 500 ppm solution was prepared by dissolving the proper amount of CuSO_4_, in ultrapure water. Metal adsorption tests were performed by stirring 30 mg of citric NS (from different synthesis, TSE, and batch) in 10 mL of colored metal solution. At the initial time and after 24 h (the time was chosen because after 24 h, there is the max of absorption with this system) (Rubin Pedrazzo et al. 2019). The dispersions were centrifuged (for 10 min at 4000 rpm) and the supernatant filtered using 0.2-μm PTFE syringe filters and analyzed by UV-Vis (at 830 nm) using a Perking Elmer UV/Vis Spectrometer Lambda 25 for quantifying the residual uncomplexed metal. Adsorption was carried out at RT and under continuous stirring.

### Adsorption of methylene blue (≈ 2.6 ppm)

A ≈ 10^−5^ M methylene blue solution was prepared by dissolving the proper amount of organic dye, in ultrapure water. Metal adsorption tests were performed by stirring 10 mg of each NS in 10 mL of colored solution. After 24 h, the dispersions were centrifuged (4000 rpm, 10 min) and the supernatant was filtered using 0.2 μm PTFE syringe filters.

## Results and discussion

**Fig. 1:**
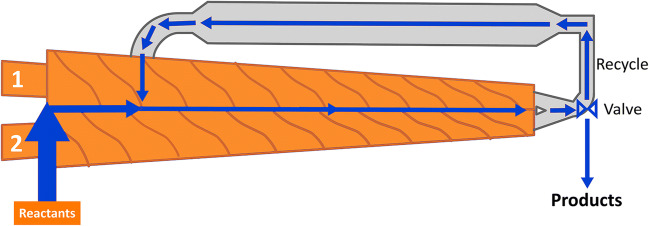
Schematic representation of a lab scale twin-screw extruder. Reactants are inserted between the two preheated screws in a closed chamber. After a first mixing it is possible reintroduce the material in the screw chamber

All the polymers synthetized, both using “classic” batch method and Twin-Screw method are indicated in Table 1. The abbreviation βNS-Citr 1:4 TSE refers to a cross-linked β-cyclodextrin-based polymer (NS) and obtained by cross-linking citric acid, twin-screw extruder (TSE). The number following the cross-linker in the abbreviation refers to the molar ratio between the cyclodextrins and cross-linker. The same notation is used NS obtained from batch. Two different ratios (1:4 and 1:8) were tested using the β-cyclodextrin for both methods. The simplified structure of the extruder is reported in Fig. 1

**Table 1:** Summary table. Samples are reported with synthesis conditions, adsorption results (ads/tot [%]), ζ potential, BET surface area (m^2^/g)

**Sample name**	**Synthetic method**	**Reaction conditions**	**ζ potential**	**BET surface area [m**^**2**^**/g]**	**Adsorption tests**	
					**CuSO**_**4**_	**Methylene blue**
					**Cu**^**2+**^_**ads**_**/Cu**^**2+**^_**tot**_ **[%]**	**MB**_**ads**_**/MB**_**tot**_ **[%]**
βNS-Citr 1:4	Batch	140 °C for 1 h, 100 °C for 4 h under vacuum ~ 20 mbar	− 21.8 ± 3.10	1.76 ± 0.04	58.3	74.3
βNS-Citr 1:8		140 °C for 1 h, 100 °C for 4 h under vacuum ~ 20 mbar	− 29.4 ± 11.1	1.30 ± 0.05	64.5	84.3
βNS-Citr 1:4 TSE	Twin-screw extruder	150 °C for 15 min	− 33.8 ± 4.29	1.10 ± 0.06	79.9	83.5
βNS-Citr 1:8 TSE		150 °C for 15 min	− 26.0 ± 6.85	0.93 ± 0.03	71.02	75.7

Basically, a Lab scale TSE consist of a closed chamber containing two rotating screw and a recycle sub-chamber. It is possible to mix and to heat at the same time. Before each experiment, a calibration of the temperature and of the applied shear/stress is performed. The feed for the extruder, that in this case is the mixture of reactants, are inserted directly between the rotating screws. The volume (mL) of feed is, for all samples, approximately around 10 mL (recycling chamber plus screws volume). The instrument is preheated at 150 °C, melting point of citric acid, and the solid mixture is inserted slowly inside the instrument. The reaction occurs usually in a short time (15 min): the progress of the reaction can be kept under control thanks to the force sensor that controls the shear applied by the two screws. When the cross-linking occurs, there is an immediate raise of the applied stress. After cross-linking a dried powder is obtained. The powder is extruded or recovered from the recycle chamber of the TSE.

As usual for mechanochemistry (Wang 2013; Jicsinszky et al. 2017a), the synthetic procedure was fast to carry out and gave for all samples a good yields (≈ 65%). The yield was calculated by considering the weight only of the insoluble part of the dried polymer. The obtained yield, even if interesting, is lower if compared with the classic batch synthesis due to the difficulty in removing small quantities from an instrument originally designed for continuous production of liquid/melt materials. The actual yield needs therefore to be calculated with a similar set-up but on a scaled-up reaction, with larger amounts of reactants and product. Furthermore, the possibility of working with a recycling of the unreacted material should be considered.

The reaction time is considerably shorter. Since the reaction of cross-linking is a polycondensation that leads to a polyester, water needs to be eliminated from products for enhancing the kinetic of the reaction: this can be achieved by heating and by working using a vacuum pump. The whole amount of water necessary for solubilizing all reactants needs to be eliminated, and this is not necessary for a solvent-free approach. Moreover, the application of a continuous shear during all reaction permits an easy activation of the chemical bond fastening the kinetic: the reaction is not achievable at the same conditions without the application of mechanical forces.

The solubility in different common solvents (acetone, ethanol, N,N-dimethylformamide, dimethylsulfoxide, diethyl ether, petroleum ether, and water) of the new nanosponges was tested: NSs from twin-screw extruder are insoluble in the tested solvents, in accordance with the formation of an actually cross-linked network and with data from previous literature (Trotta 2011; Trotta and Rubin Pedrazzo 2021).

The zeta potential of colloidal suspensions of βNS-Citr from TSE was tested and compared with “classic” NSs. In general, it is possible to say that the stronger the charge, the better is the colloidal stability of the particles. As shown in Table 1, all CD Citr polymers exhibit a negative ζ-potential, and this is coherent with previous literature (Trotta 2011; Trotta et al. 2011; Dhakar et al. 2019). The negative is usually related to the molar ratio of cross-linker: the larger the amount of Citric acid, the more negative the ζ-potential detected. The measured ζ-potential is coherent with the amount of citric acid except for what concern the βNS-Citr 1:4 TSE that shows a surprisingly negative zeta potential: we experienced the same situation in case of other mechanosynthesis, and since the higher stability of suspensions achieved, this is desirable. Moreover, this is also confirmed by the high adsorption of methylene blue, organic dye bearing a positive charge. However, it must be considered that the possibility that not all negative groups can easily be accessible in the lattice, and this can affect both absorption and zeta potential.

**Fig. 2:**
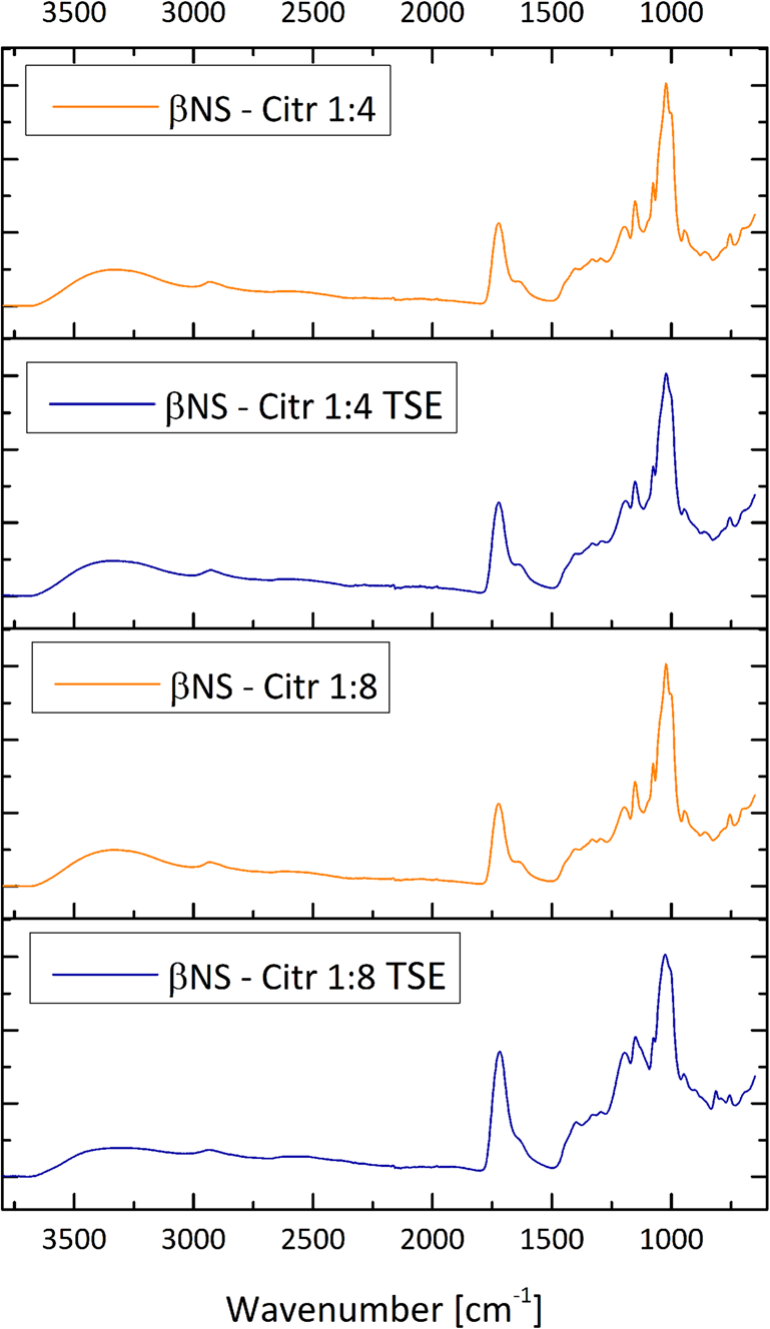
Fourier transform infrared analysis in attenuated total reflectance mode (FTIR-ATR) of βNS-Citr 1:4 and 1:8, comparison of samples from different synthetic pathway. Spectr in orange “classic” solvent synthesis, in blue TSE synthesis

**Fig. 3:**
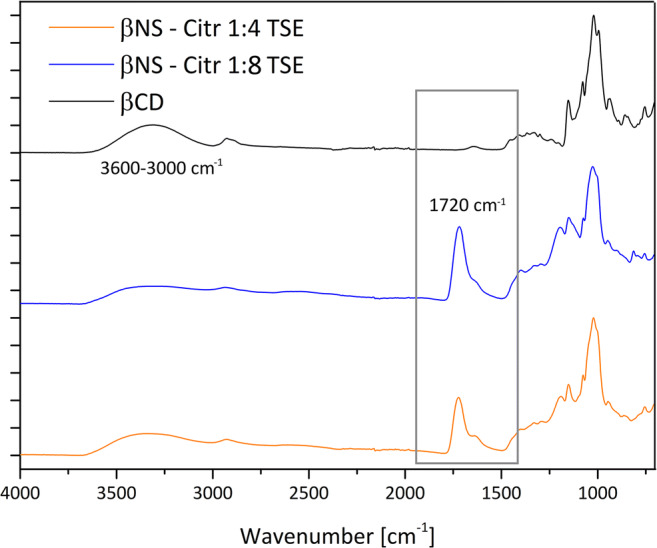
Fourier transform infrared analysis in attenuated total reflectance mode (FTIR-ATR) of βNS-Citr 1:4 and 1:8, from Twin-Screw Exruder, compared with plain β cyclodextrin, showing the presence of a peak at 1720 cm^−1^ attributable to the presence of a carbonyl group and to the formation of an ester bond

The synthesized dextrin polymers were characterized by means of infrared and thermogravimetric analysis. The infrared spectra of the citric acid NSs from TSE are reported in Fig. 2. A wide absorption band, related to the stretching of O–H bonds, visible in all cyclodextrin and cyclodextrin polymers, is observed in the 3600–3000 cm^−1^ range. More interesting is the strong absorption peak that appears at 1720 cm^−1^ that is attributable to the stretching vibration of the C=O bonds of the carboxyl groups of citric acid molecules and the ester bonds between citric acid and cyclodextrin units. As shown, this peak is not present in the CD spectrum, whereas is present in both TSE Citric polymers. The peaks in the region 1200–1000 cm^−1^ region are mainly related to the stretching vibrations of C–O bonds of ether and alcohol groups of βcyclodextrin and citric acid units. In Figs. 2 and 3 a comparison between βNS-Citr 1:4 and 1:8 is reported, from solvent synthesis and from twin-screw extruder and a comparison of βNS-Citr 1:4 and 1:8 TSE and pristine βCDs.

The spectra recorded from the same polymer but from different synthetic approach are nearly superimposable, clearly exhibiting at about 1720 cm^−1^, discussed above.

**Fig. 4:**
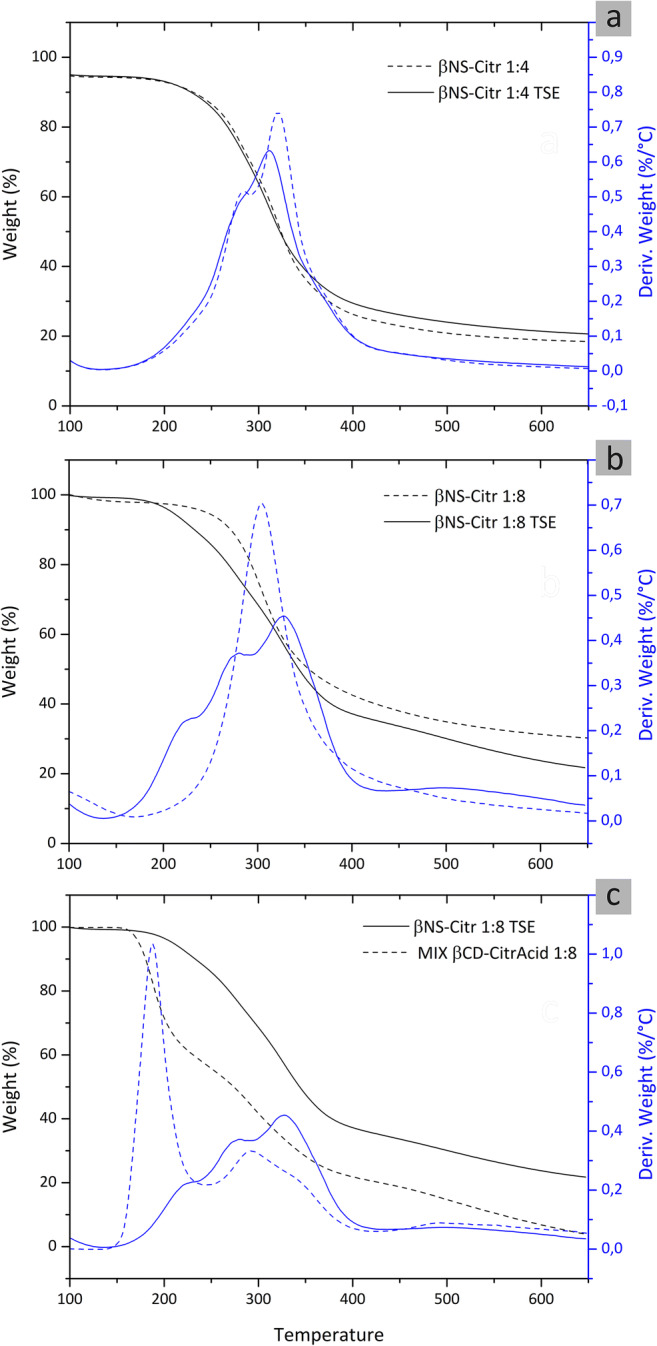
Thermogravimetric analysis of β-CD-based citric nanosponges, from TSE (straight lines) and solvent synthesis (dash lines). TG analysis curves in black, and DTG curves in blue. **a** βNS-Citr 1:4/βNS-Citr 1:4 TSE. **b** βNS-Citr 1:8/βNS-Citr 1:8 TSE. **c** Comparison between βNS-Citr 1:8 TSE and physical mixture of βCD and citric acid (no reaction). Nitrogen flow, ramp rate 10 °C/min, r.t. to 700 °C

Figure 4 reports the thermal degradation of βNS-Citr 1:4 and 1:8, in three different comparison between classic and TSE NSs and a simple physical mixture of βCD and citric acid, with the same molar ratio of a βNS-Citr 1:8. Both TG and DTG curves are reported.

The comparison between βNS-Citr 1:4 and βNS-Citr 1:4 TSE, Fig. 4a, shows two almost superimposable degradation path; consequently, the same molecular structure is expected.

More specifically, the degradation of the two cross-linked materials starts at about 200 °C and occurs in one single step, leaving a carbonaceous residue, around 20%/wt, which is thermally stable, decomposing at a very low rate at higher temperatures. A different situation is evidenced by the comparison of curves between βNS-Citr 1:8 and βNS-Citr 1:8 TSE, Fig. 4b, especially in the DTG analysis: a visible peak in the DTG at around 200–210 °C is evidenced. This peak is related to a small weight loss that starts at about 150 °C; since the reported Mp of the citric acid is 150 °C, we assumed the presence of free citric acid or the formation of CA “oligomers” and/or CA short chains bonded to the NSs structure: to confirm and support this hypothesis, we performed a TG analysis comparing βNS-Citr 1:8 TSE and a physical mixture of citric acid and βCD, with the same molar ratio (Fig. 4c) evidencing the presence in the DTG of the same peak. Whereas is clear from Fig. 4, that the step attributable to the citric acid is considerably lower, confirming the formation of a cross-linked polymer. It is possible to presume that the reaction of the βNS-Citr 1:8 TSE, occurring in significantly short times and in presence of a high quantity of cross-linker, could not allow the formation of a completely cross-linked material, leading also to the formation of short CA chains bonded to the NS insoluble structure.

It is anyway evident that the degradation path of βNS-Citr 1:8 and βNS-Citr 1:8 TSE is quite similar, especially for what concern the major loss of weight (related to the thermal degradation of the βNS-Citr structure).

BET data are reported in Table 1. All BET surface area values [m^2^/g] are comprised between 1 and 2 m^2^/g, the different preparation method leads to a very modest reduction of the surface areas, whereas a different content of citric acid leads to a slight increase in the area.

### Water absorption capacity (WAC)

**Fig. 5:**
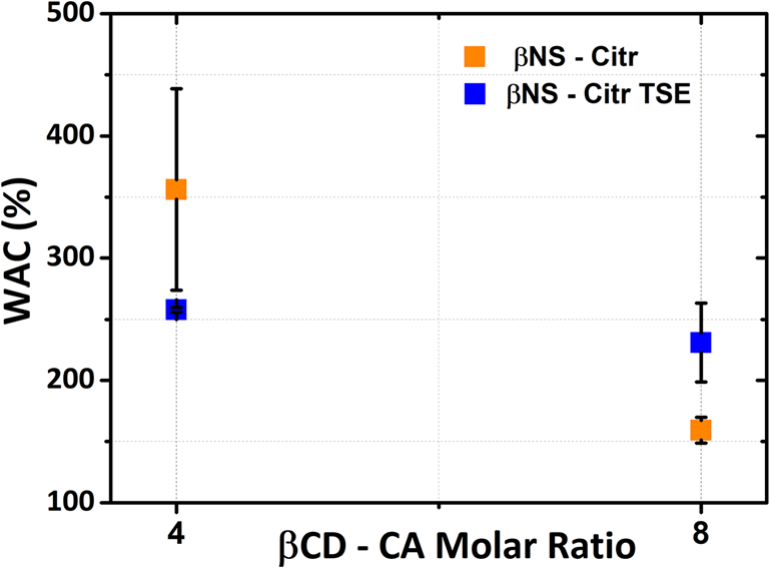
Water absorption capacity (WAC) as a function of cross-linker to monomer ratio of βNS-Citr

The βNS-Citr batch syntheses exhibited higher water absorption capacity (WAC) than the βNS-Citr TSE syntheses. The WAC values are greater for 1–4 βNS-Citr syntheses, in two methods, compared to the 1–8 βNS-Citr syntheses, as presented in Fig. 5. Experimental values in Table 2 show that the WAC of βNS-Citr powders is between 150 and 360%. As the molar ratio increases, the WAC decreases due to the restrictions of the movement of polymer chains. This makes the structure more compact and hampers the diffusion of water in the polymer network (Hoti et al. 2021; Tavera-Quiroz et al. 2018). This confirms what is already observed in the literature (Lee et al. 2018), the swelling ratio decreases when the amount of citric acid as a cross-linker increases. This is because of strong cross-linking formation by the ester bridge between sugar monomers and citric acid, interfering with water penetration. There is a slight difference of the swelling ratio in the case of molar ratios of twin-screw extruder synthesis. When comparing the results of both methods, a decrease in the WAC is observed for molar ratio 1:4 of TSE synthesis. Obviously, the swelling decreases as the reaction temperature is increased from 100–140 up to 150 °C, irrespective of the reaction time, confirming what has been analyzed before (Petitjean et al. 2020), whereas we have the opposite situation for molar ratio 1:8 of TSE synthesis.

**Table 2:** WAC experimental values of βNS-Citr as a function of the cross-linker content

**βNS-Citr molar ratio**	**Batch synthesis**	**Twin-screw extruder**
1–4	356 ± 82.27	258 ± 1.89
1–8	159 ± 10.6	231 ± 32.33

### Cross-linking density determination using Flory-Rehner theory

**Fig. 6:**
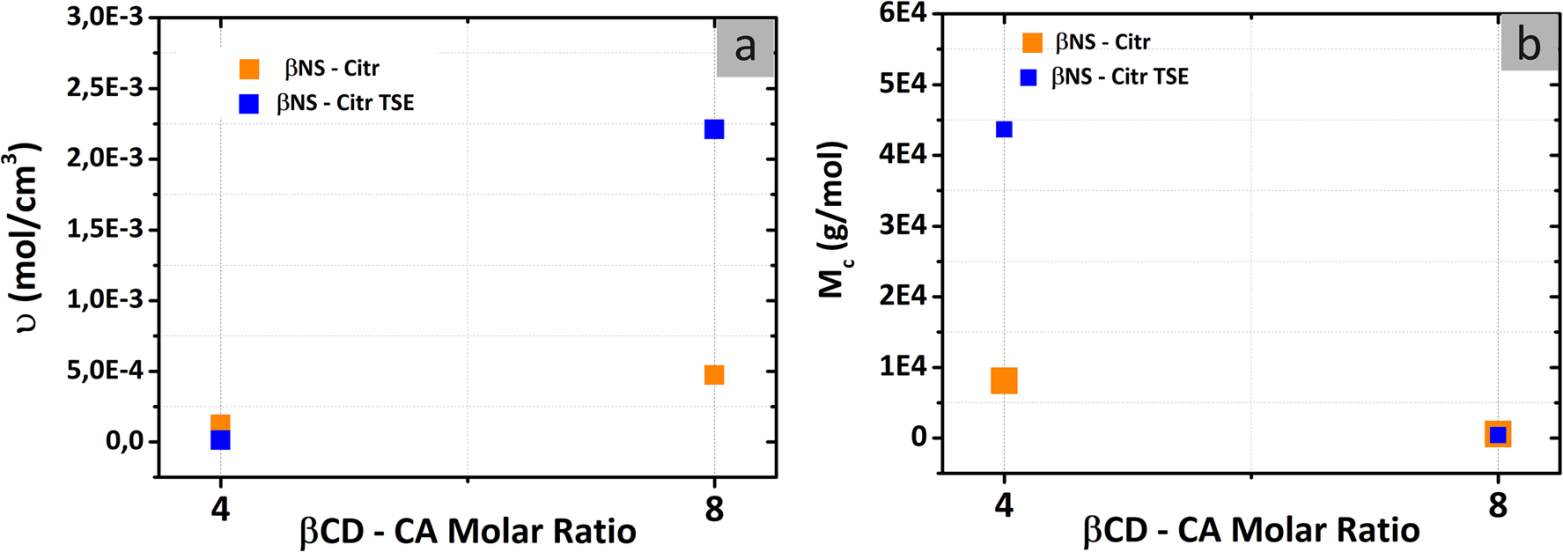
**a** Cross-linking density υ (mol/cm^3^) and **b** molecular weight between cross-links M_c_ (g/mol) from the equilibrium swelling of prepared βNS-Citr

Figure 6a shows that by increasing the molar ratio, the cross-linking density increases as well. βNS-Citr batch syntheses, compared to βNS-Citr TSE syntheses, show a similar cross-linking density in the case of molar ratio 1: 4. Contrariwise, molar ratio 1:8 synthesized with TSE, at 150 °C, shows a higher cross-linking density. This is in agreement with the literature (Ma et al. 2018), where the cross-linking density increases with the cross-linking temperature. Notably, cross-linking conditions, as described earlier, alter the cross-linking density. Further, at higher cross-linking density, the average distance between two cross-link points (M_c_) becomes shorter and the network becomes denser. Therefore, with the decreasing of the cross-linking ratio in βNS-Citr the experimental values of M_c_ increase, as presented in Fig. 6b. To meet the requirements of a specific application, it is a predominant strategy to control the cross-linking degree (υ) or molecular weight between cross-links (M_c_). Then, the mechanical properties are tuned accordingly (Tran et al. 2015).

### Rheological measurements

**Fig. 7:**
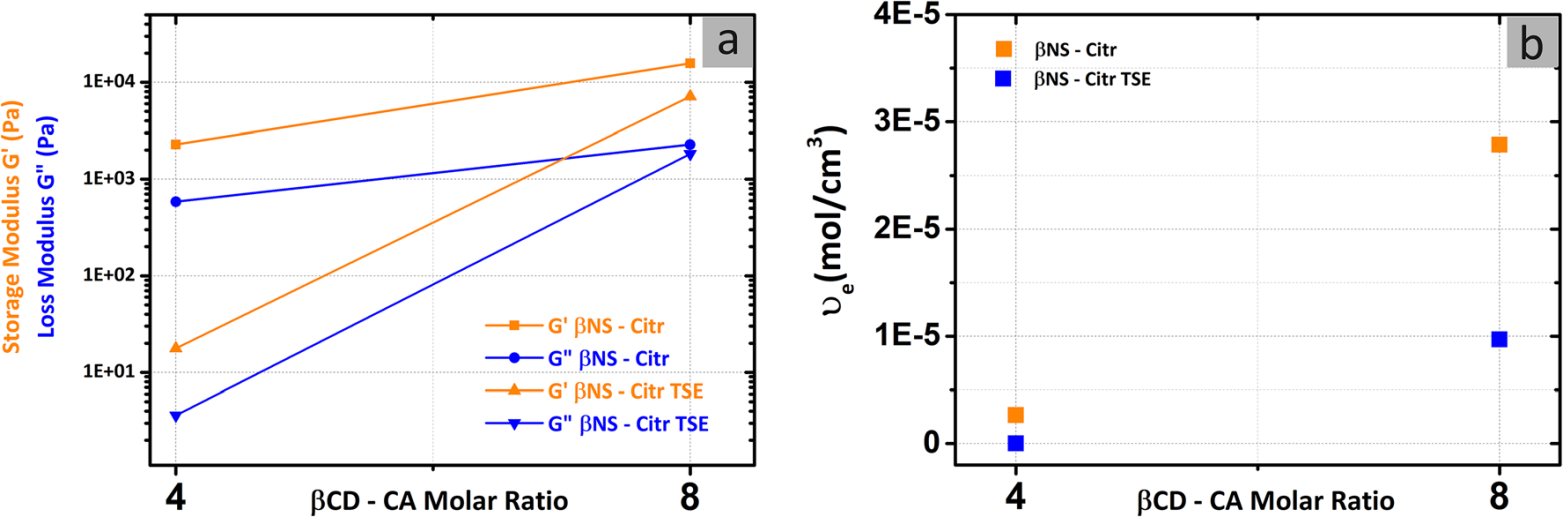
**a** Storage (G’) and loss (G”) modulus versus molar ratio of βNS-Citr (1–4; 1–8) at an angular frequency (ω) of 1 rad/s. **b** Effective sub-chain density (moles of effective sub-chains per unit volume, υ_e_, mol/cm^3^) as a function of added cross-linker content

A variation of the cross-linker content and cross-linking conditions, according to literature, can affect the mechanical properties of βNS-Citr (Ma et al. 2018). With this in mind, Fig. 7A presents the viscoelastic behavior of the synthesized βNS-Citr as a function of an angular frequency (ω) of 1 rad/s. It is showed that the powders of both methods are similar in the profile of storage modulus (G’) and loss modulus (G”), where the values for TSE are lower. The G’ is higher than G”, in both methods (βNS-Citr: 1–4; 1–8 + TSE), verifying the appearance of the material as a viscoelastic gel. The storage modulus (G’) is used to calculate the number of effective chains per unit volume (υ_e_), defined as chains connected at both ends to cross-links. Figure 7B shows that by increasing the cross-linker content, the υ_e_ increases as well, confirming what was already observed in the previous article (Hoti et al. 2021). The comparable results from batch synthesis and TSE present the values of G’, G”, and υ_e_ in the case of TSE, being consistent with a study published earlier (Alam et al. 2020). The gel becomes weaker because the elastic moduli decrease (leading to the decrease of the υ_e_) at higher temperatures.

To sum up, the extrusion process just affects the mechanical properties of βNS-Citr but does not alter its structure. The modification may be due to different mechanical energy input of TSE; this is in agreement with findings from a previous work (Lammers et al. 2018).

By tuning the synthesis conditions and properties such as mobility of the network chains and mechanical rigidity, the gel structure can be considered a hydrogel that can be used in drug delivery and tissue engineering (Salimi-Kenari et al. 2018) and microfluidic applications (Johnson et al. 2004).

**Fig. 8:**
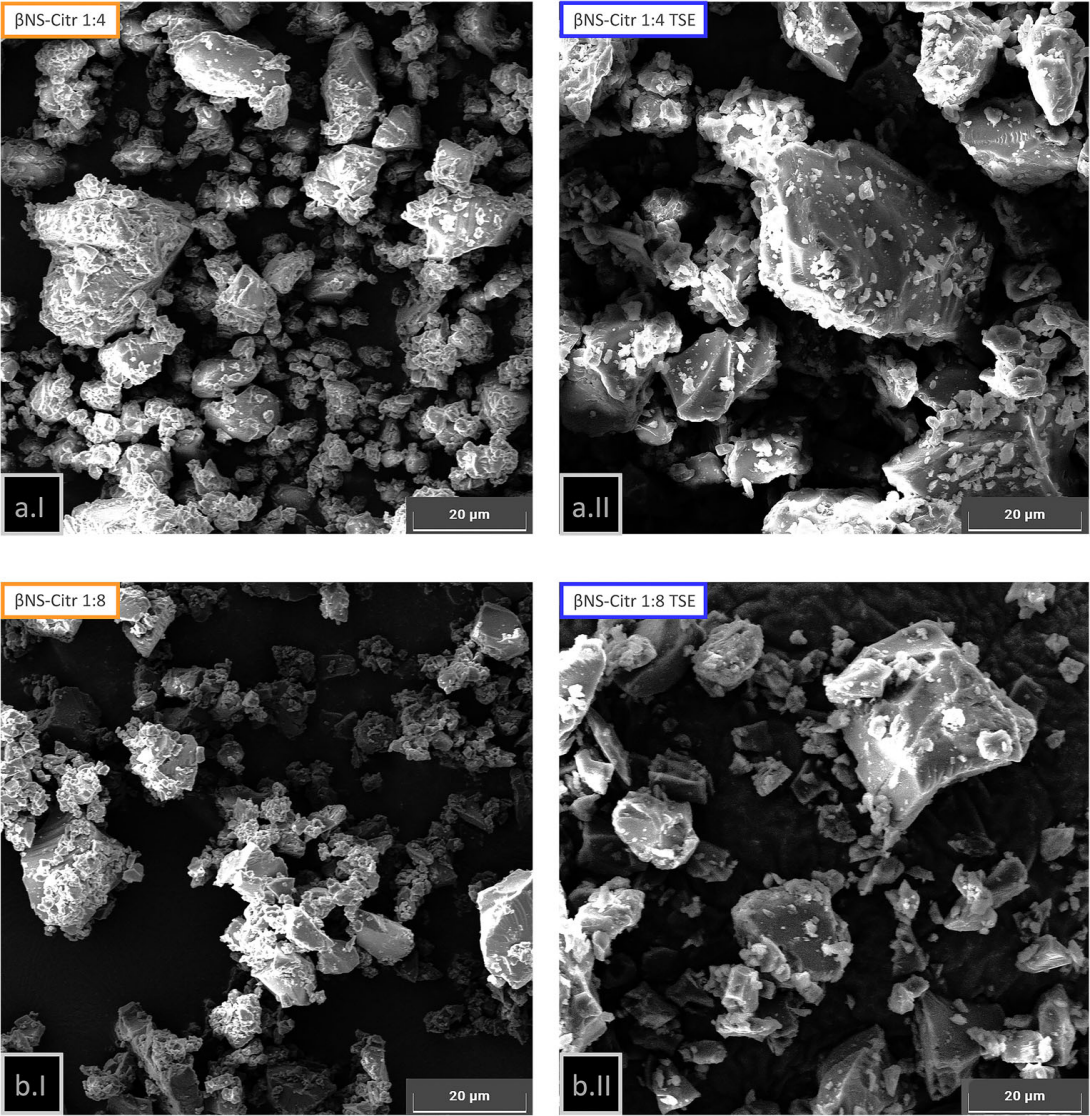
FE-scanning electron micrographs: βNS-Citr 1:4 from batch (**a.1**) and TSE (**a.II**); βNS-Citr 1:8 from batch (**b.1**) and TSE (**b.II**)

The overall morphology and particle size of the NSs were evaluated using SEM analysis. All NSs, from both batch and TSE syntheses exhibited irregular morphology and broad size distribution in the micrometer range (Fig. 8). As already said the average particle size of all samples is comparable (≥ 800 nm, via DLS). Such particle size is quite desirable for environmental applications, since it allows fast precipitation and easy separation of the NSs from the treated solution. It is worth to say that the reported DLS technique value refers to an average; as clearly visible in FE-SEM images (Fig. 8, following), particles, especially for what concern TSE syntheses, exhibit heterogeneous sizes.

The granulometry of samples deserves a separate discussion: the use of ball mill for particle size reduction is necessary, as permit comparable powders, for granulometry and particle size, to be obtained. The shear of the balls anyway dramatically changes the external shape of the small grains. Anyway, even after the ball mill process, we can notice a difference in the overall granulometry of the powder: it is showed from FE-SEM images that, in general, both from 1:4 and 1:8 molar ratio, powder from batch synthesis exhibit smaller particles or, more specifically, aggregates of small particles of similar size. The results are coherent with what previously said: a more “brittle” mechanic behavior is assumable from βNS-Citr batch. Absorption performances of βNS-Citr batch and TSE are not affected by the morphology of the powder, as will be shown in the next paragraphs.

### βNS-Citr TSE for water remediation

#### Adsorption of Cu^2+^ ions (≈ 500 ppm)

**Fig. 9:**
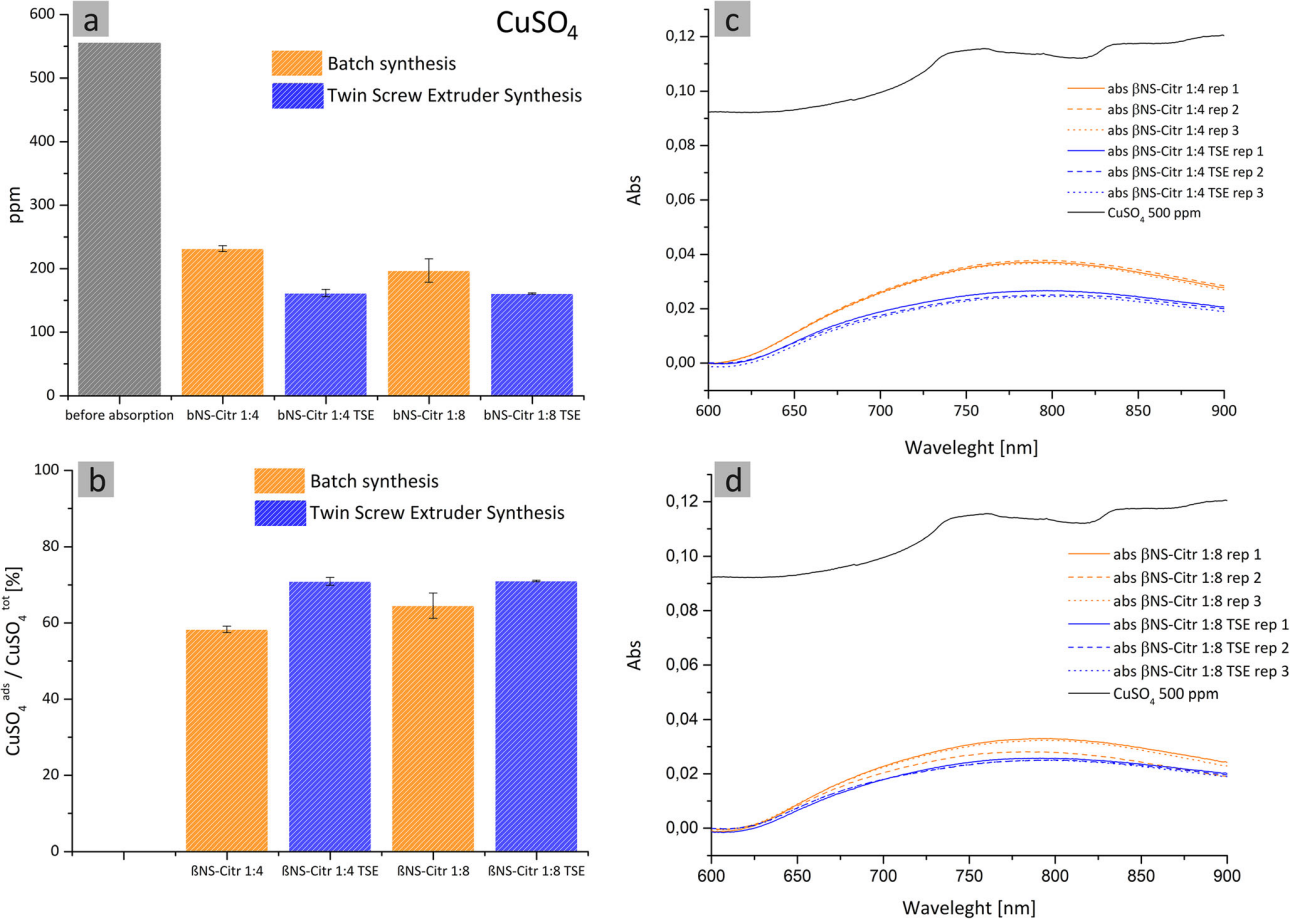
Metal adsorption tests performed in 500 ppm metal solutions. The NSs adsorption capacity is expressed as a percentage of the removed amount of metal (**b**) and ppm before/after absorption (**a**). UV spectra of βNS-Citr 1:4 (batch and TSE) (**c**) and βNS-Citr 1:8 (batch and TSE) (**d**) are reported

The capacity of the new synthetized NSs of adsorbing heavy metals from highly concentrated metal solutions (500 ppm) was evaluated. Figure 9b shows the amount of complexed metal ions in water before and after 24 h as a percentage of the adsorbed amount, in inset a is reported the concentration of Cu^2+^ (ppm)

The NSs complexed quantity of metal ions is comprised between 50 and 80% for all samples, slightly higher for the samples from TSE synthesis. The higher adsorbed quantity is coherent with the higher swelling.

#### Adsorption methylene blue (stock solution ≈ 10^−4^ M, ≈ 2.60 ppm)

The same experiment was performed, with the same modalities and amounts of polymer, using methylene blue as probe molecule.

**Fig. 10:**
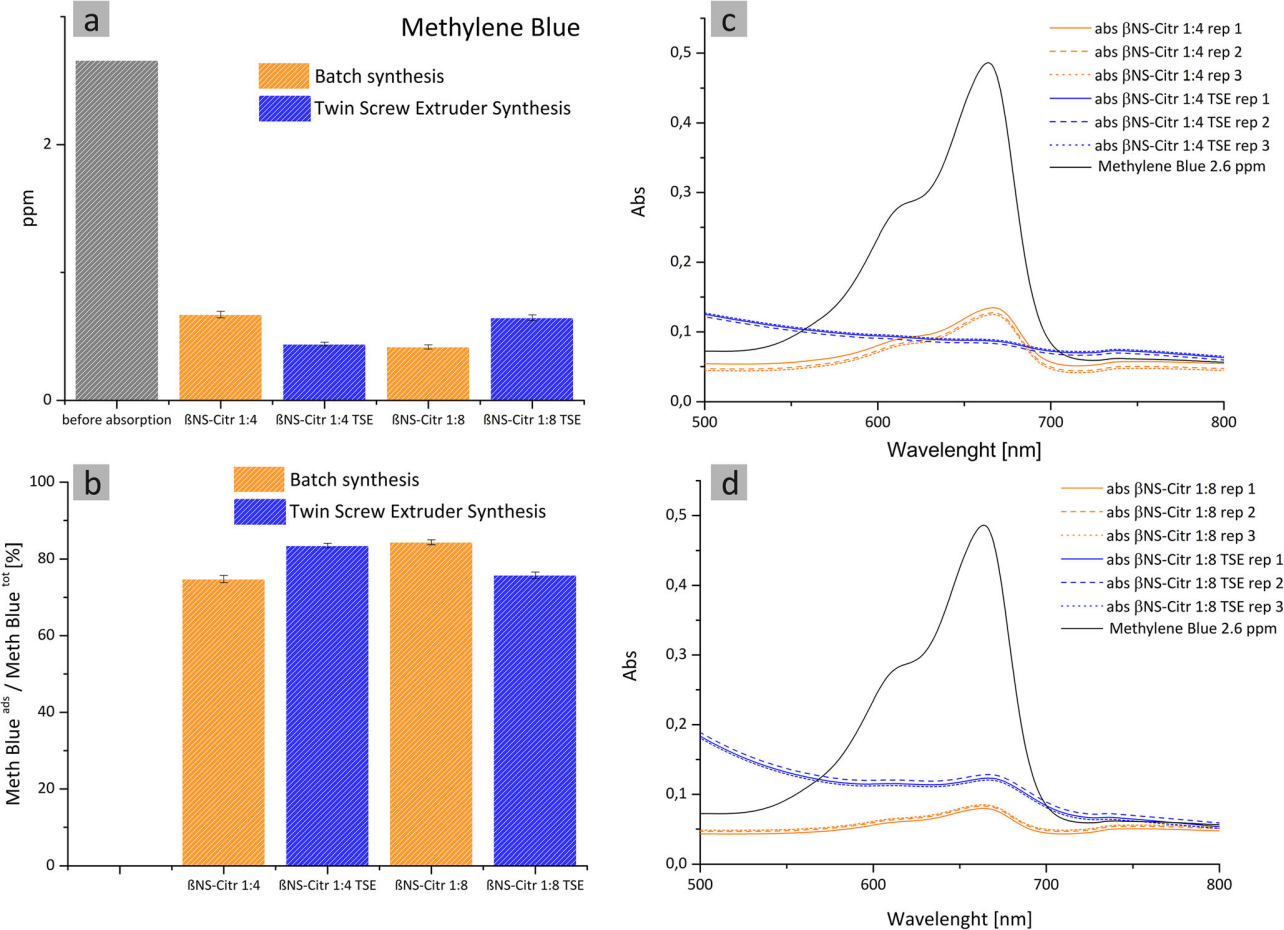
Methylene blue adsorption tests performed in 2.6 ppm dye solutions. The NSs adsorption capacity is expressed as a percentage of the removed amount of organic dye (**b**) and ppm before/after absorption (**a**). UV spectra of βNS-Citr 1:4 (batch and TSE) (**c**) and βNS-Citr 1:8 (batch and TSE) (**d**) is reported

The choice fell on methylene blue because the molecule bears a positive charge and because is a widely known and used dye (Zhang et al. 2003; Guo et al. 2013; Jiang et al. 2019). Results are reported in Fig. 10.

As shown, in this case, there is no visible trend. The solution is, as said, diluted, so the adsorbed quantities are as expected high and always comprised between 70 and 90% (MB_ads_/MB_tot_ [%]). The best results in complexation of MB are achieved by the sample βNS-Citr 1:4 TSE: this is coherent with the highly negative zeta potential that can interact with the positive charge of the polymer.

## Conclusions

βCD-Citr nanosponges, with the same characteristics as cyclodextrin-based polymers synthesized using solvent in batch, were obtained without the use of any solvents, via a mechanochemistry based synthesis.

The reaction here reported is totally driven using a twin-screw extruder (TSE), as a chemical reactor. TSE has recently shown a great potential in the continuous organic synthesis and is extensively used for the reactive extrusion of polymers, permitting a fine temperature control and a continuous process. The mechanosynthesis permitted us not to use any solvents and to reduce the reaction time from more than 4 h to 15 min.

The obtained polymer exhibited the same physio-chemical characteristics of the polymer synthetized in solvent: insolubility, swelling properties, and complexes formation.

Since a new synthetic method is here introduced, we opted for a quite comprehensive and methodical characterization, with different techniques, to better understand the characteristics of the material.

We demonstrated the formation of an insoluble lattice and the new materials were characterized via FTIR and TGA (comparing the thermal stability of the two different synthetized materials) and the WAC and Flory-Renner theory confirmed the behavior of the material in presence of water.

This specific type of absorbent is particularly promising for water remediation, since it is biocompatible and biobased, and the insolubility permits an easy recovery of the absorbent and absorbate. Moreover, the possibility to synthetize βNS-Citr with a continuous method, efficient and faster than a batch approach, is quite promising for these types of application where huge volumes of materials are usually involved.

Therefore, the obtained NSs were tested for the adsorption and removal of Cu^2+^ (CuSO_4_ solutions) and methylene blue (MB) from aqueous solutions, in comparison with NSs prepared by cross-linking β-CD with CA in batch, showing comparable, if not superior adsorption.

## Supplementary Information

ESM 1 (PDF 475 kb)
