# Supplementary material for: Sustainable mechanochemical synthesis of β-cyclodextrin polymers by twin screw extrusion
Source: Environ Sci Pollut Res Int. 2021 Aug 23;29(1):251–63. doi: 10.1007/s11356-021-15187-5 (PMC8724137; doi:10.1007/s11356-021-15187-5)
Supplement: Supplementary file 1 — (PDF 475 kb) [file 11356_2021_15187_MOESM1_ESM.pdf]

SEM (βNS-Citr 1:4 TSE)

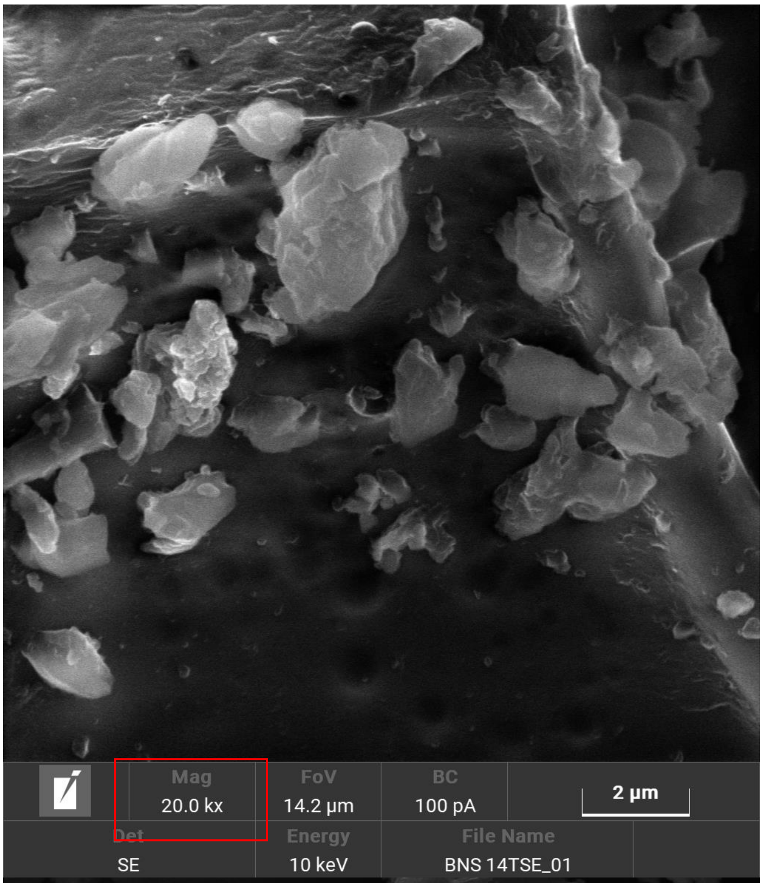

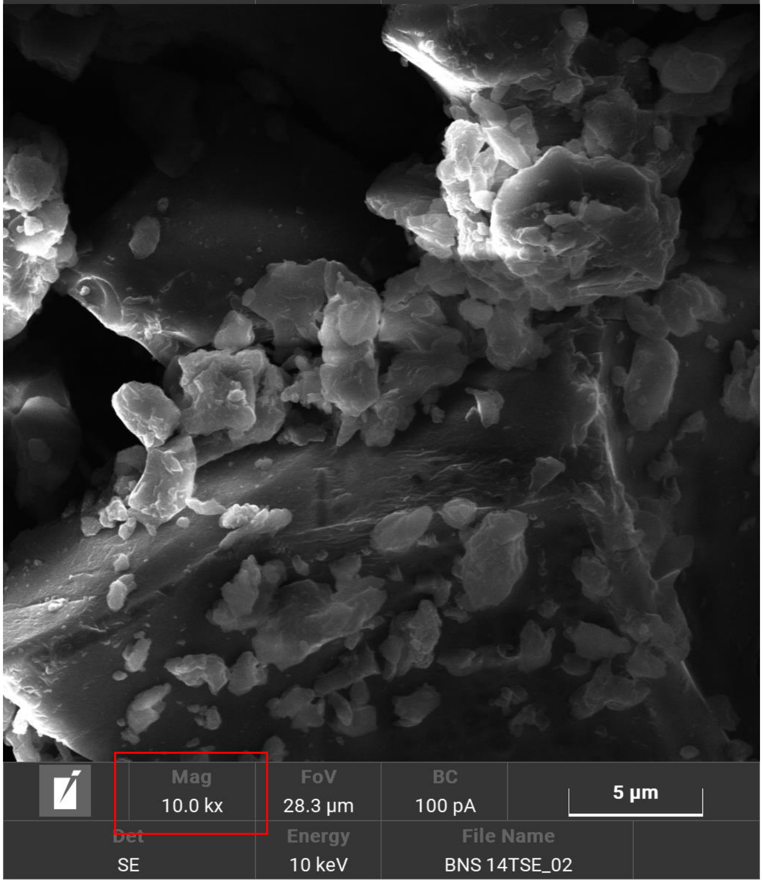

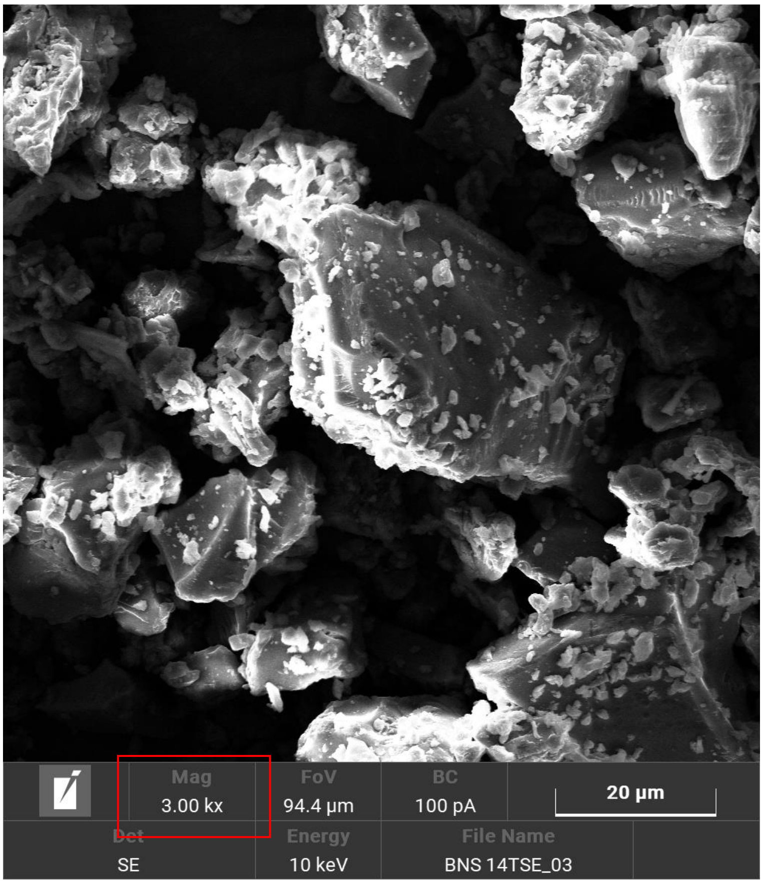
Mag
3.00 kx
FoV
94.4 µm
BC
100 pA
20 µm
Det
SE
Energy
10 keV
File Name
BNS 14TSE_03
